# The effect of three-dimensional computed tomography reconstructions on preoperative planning of tibial plateau fractures: a case–control series

**DOI:** 10.1186/s12891-015-0608-0

**Published:** 2015-06-13

**Authors:** Andrew Dodd, Elizabeth Oddone Paolucci, Robert Korley

**Affiliations:** Orthopedic Surgery Residency Training Program, University of Calgary, Calgary, Alberta Canada; Office of Surgical Research, University of Calgary, Calgary, Alberta Canada; Division of Orthopaedics, University of Calgary, Calgary, Alberta Canada; Division of Orthopaedic Surgery, Health Sciences Centre, 3330 Hospital Drive NW, Calgary, AB T2N-4N1 Canada

## Abstract

**Background:**

Tibial plateau fractures are a common intra-articular injury for which computed tomography (CT) scans are routinely used for preoperative planning. Three-dimensional reconstructions of CT scans have been increasingly investigated in recent years, however their role has yet to be defined. We wish to investigate the role of three-dimensional computed tomography reconstructions (3D-CT) in the preoperative planning of tibial plateau fractures.

**Methods:**

Twelve cases of tibial plateau fractures including plain film radiographs and conventional CT scans were distributed to 21 observers (orthopaedic residents and consultants). The observers filled out a preoperative plan checklist created for this study. Three months later the same cases were distributed, in random order, this time including 3D-CT reconstructions. The same preoperative checklists were completed, and compared to the previous checklists.

**Results:**

The preoperative plan checklist was able to detect differences between cases and between observers. No significant differences were detected between the total plan scores when comparing conventional CT to 3D-CT. Sub-analysis of plan specifics (incisions, hardware, adjuncts) was also not significantly different. The level of training of the observer or the fracture complexity did not affect these results.

**Conclusions:**

No significant changes were made to observer’s preoperative plans with the addition of 3D-CT. 3D-CT reconstructions come at a cost to the system, and therefore their usefulness should be investigated prior to widespread use. Our study demonstrates that the addition of 3D-CT reconstructions to the preoperative workup of tibial plateau fractures did not change management plans when compared to plans made using traditional CT-scans.

## Background

The advent of computed tomography (CT) with three-dimensional reconstructions (3D-CT) has been a topic of increasing investigation recently. Most research focuses on the influence of 3D-CT on fracture classification systems [1–9]. The results of these studies have varied, however there is a trend towards improved intra- and inter-observer reliability of fracture classification systems when three-dimensional reconstructions are used. Other authors have investigated the influence of 3D-CT on the management of specific injuries [5–7]. These previous studies have focused on the accuracy of preoperative plans compared to the final surgical tactic, or on the inter- and intra-observer reliability of preoperative plans. Few authors have investigated whether or not the addition of 3D-CT changes a preoperative plan that was made based on traditional two-dimensional CT reconstructions (2D-CT).

Tibial plateau fractures are a common intra-articular injury for which a CT-scan is routinely ordered [10–12]. CT-scans have been shown to improve the intra- and inter-observer reliability of fracture classification and management plans in the setting of tibial plateau fractures [13, 14]. Chan *et al.* demonstrated a large change in preoperative plans with the addition of CT scans to plain radiography. Currently there is little data on the influence of 3D-CT on preoperative planning for tibial plateau fractures. The goal of the current study is to examine whether the addition of 3D-CT to 2D-CT and plain radiography results in a significant change to the preoperative planning of tibial plateau fractures. Our hypothesis is that the addition of 3D-CT will have minimal impact on preoperative plans when compared to 2D-CT.

## Methods

### Study design

Ethical approval was obtained from the Conjoint Health Research Ethics Board (CHREB) at the University of Calgary. The CHREB agreed to waive informed consent as no direct patient care or records were involved in the study. Subjects radiographs and CT scans were accrued as a subgroup of those currently involved in another study examining tibial plateau fractures. Subjects were selected randomly from this group, with the goal of collecting a group that is representative of local practice. No authors have financial or other conflicts of interest to declare with regards to this study.

Inclusion criteria included patients greater than 18 years of age who suffered a traumatic tibial plateau fracture that necessitated surgical management, and who had a CT-scan ordered for preoperative planning. Exclusion criteria included patients less than 18 years of age, periprosthetic fractures, insufficiency or pathological fractures, and metabolic or genetic disorder affecting bone.

The fractures were assessed by four senior trauma-trained staff orthopaedic surgeons at a level I trauma centre, nine senior orthopaedic residents (PGY-4 and 5), and eight junior orthopaedic residents (PGY-2 and 3) for a total of 21 observers. Each observer was given a study package of 12 cases. The first package included plain radiographs and standard CT-scans with two-dimensional reconstructions in the axial, coronal, and sagittal planes (2D-CT). A preoperative planning checklist (Table 1) was included in the package. Each observer filled out the preoperative planning checklist for each case. Three-dimensional surface-rendered CT reconstructions were then made using Osirix software (freeware, Pixmeo – Geneva, Switzerland) by one of the investigators. The three-dimensional reconstructions were able to be manipulated 360 degrees in the coronal plane. The distal femur and proximal tibia were included in the reconstructions. Three months after the first study package was completed, a second study package was distributed which included plain radiographs, 2D-CT, and 3D-CT of the same cases. Cases were presented in a randomized order compared with the first study package. Each observer completed the same preoperative planning checklist for each case, this time having access to the 3D-CT images.

**Table 1:** Preoperative planning checklist

Preoperative Plan - Check all applicable for definitive management		
Technique	Minimally Invasive Plate Osteosynthesis (no arthrotomy)	
	Open Plating (arthrotomy)	
Incisions	Anterolateral	
	Posteromedial	
Implants	Medial	
	Lateral	
	Anterior	
	Posterior	
	External Fixator (definitive treatment)	
Adjuncts	Bone Graft – allograft	
	Bone Graft – autograft	
	Bone Graft – synthetic	
	Femoral Distractor	

The preoperative planning checklist was created by expert opinion and consensus as to the important aspects of the plan to capture (Table 1). The ‘technique’ section asks if the surgeon would choose to use an arthrotomy during the procedure; the concept is that more complex fractures with intra-articular comminution would be more likely to need an arthrotomy. ‘Incisions’ includes the two most commonly used incisions to approach the most common fracture patterns seen with tibial plateau fractures (anterolateral and posteromedial incisions). ‘Implants’ asks the surgeon to delineate where their plates and screws would be placed for the fracture in question, including an option for definitive external fixation. ‘Adjuncts’ considers the other commonly used tools in the operative treatment of tibial plateau fractures, such as bone graft options and use of a femoral distractor.

For data analysis, the surgical approach (incisions) and placement of implants were weighted 2:1 compared to the other aspects of the plan as these were felt to be the most significant components of the preoperative plan. Including another incision or more implants is also a more significant change in the preoperative plan than other factors. Each aspect of the plan that was checked off was scored one point, with checkmarks in the incision and hardware sections being scored two points. The score for the plan was the sum of all of the points for that particular plan, with a maximum score of 19 points.

The primary outcome was the change in preoperative plans after the addition of 3D-CT as measured by the total point scores obtained from the preoperative planning checklist for each case. Secondary outcomes include differences in preoperative plans between 2D- and 3D-CT based on training level and fracture complexity, and subanalysis of the influence of 3D-CT on each aspect of the preoperative plans (approach, incision, implants, adjuncts). Fracture classifications were determined by consensus of the authors using plain film and 2D-CT.

### Statistical analysis

Sample size was based on a two-tailed *t*-test. We used a five-point difference in mean total plan score (approximately 25 % change in plan), as we felt this represented a significant change in the preoperative plan. With a power of 0.8 and α = 0.05, a total of 468 studies, or 234 studies per group were required. With 21 observers and 12 studies, we have a total of 504, or 252 studies per group.

Frequency distribution graphs were created for all appropriate variables, and measures of central tendancy, skewness, and dispersion were calculated using standard methods.

Independent t-tests and one- and two-way ANOVA testing was performed to assess relationships between selected variables. Crosstab analyses were performed to assess relationships between categories, including the Pearson chi-squared test and Fisher’s exact test.

## Results

### Fracture classifications

The case-by-case breakdown of fracture classifications is described in Table 2.

**Table 2:** Fracture classifications

Case	Shatzker	AO/OTA
1	II	B3
2	V	C3
3	II	B3
4	II	B3
5	I	B1
6	II	B3
7	V	C1
8	I	B1
9	VI	C3
10	I	B1
11	II	B3
12	II	B3

### Sensitivity of the preoperative planning tool

The preoperative planning tool was able to detect differences between observers and between cases. The effect of the observer on the mean total preoperative plan score (mean total score) of the twelve cases combined reached statistical significance (*p* = 0.043). The case in question also influenced the mean total score (*p* =<0.001).

### Preoperative plans

The mean total score for all twelve cases in round one (2D-CT) was 6.76. The addition of 3D-CT did not significantly affect the mean total preoperative plan score (6.78, *p* = 0.936). The addition of 3D-CT did not affect the mean ‘incision’, ‘implants’, or ‘adjuncts’ point values when all twelve cases were combined (Table 3). 3D-CT did not influence the technique chosen (arthrotomy *vs.* no arthrotomy), the frequency of use of a femoral distractor, or the frequency or type of bone graft used.

**Table 3:** Mean preoperative plan scores based on 2D- and 3D-CT

	Plan type	
Plan Variable	2D-CT	3D-CT
Total Score	6.76	6.78
Incision	2.25	2.25
Hardware	2.63	2.69
Adjuncts	0.78	0.75

### Level of training

**Fig. 1:**
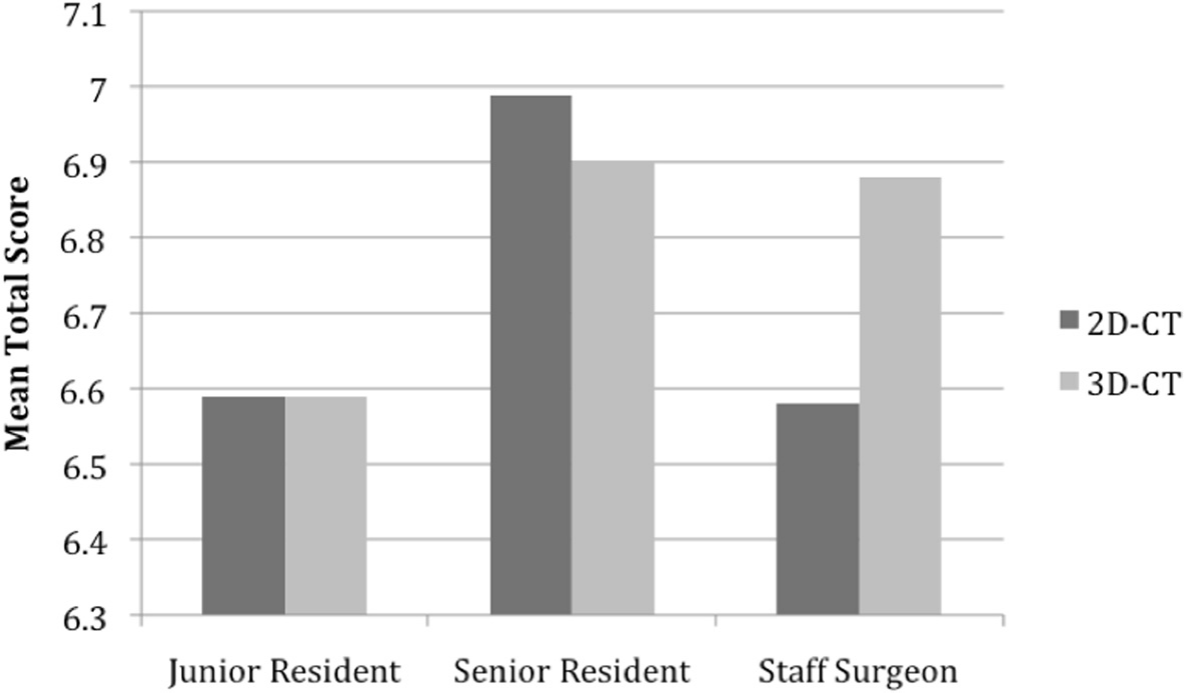
Change in mean total score from 2D-CT to 3D-CT based on level of training

The level of training of the observer did not have a statistically significant impact on the mean total score (*p* = 0.163), regardless of the type of plan (2D- vs 3D-CT). Senior residents tended to have the highest mean total scores for both 2D- and 3D-CT. The highest degree of change in plans between 2D- and 3D-CT was observed in the staff surgeon’s scores, whereas junior residents plans changed the least (Fig. 1). Despite these trends, the differences did not reach statistical significance.

The combined analysis of level of training and case number did not significantly affect the mean total scores (*p* = 0.081). Comparison between staff surgeons and residents plans demonstrated no significant differences with respect to total score, technique, or use of adjuncts. There was a statistically significant difference between staff and residents with regards to values for incision (2.17 vs 2.27, *p* = 0.01) and implants (2.79 vs 2.63, *p* = 0.027).

Residents’ plans demonstrated a statistically significant difference with regards to technique (arthrotomy *vs.* no arthrotomy) from 2D- to 3D-CT. Senior residents increased the number of arthrotomies performed from 56 % of cases to 69 % of cases (*p* = 0.049). In contrast, junior residents decreased the number of arthrotomies performed from 60 to 51 % (*p* = 0.016). Staff surgeons did not demonstrate a significant change in technique.

### Fracture complexity

Fractures were classified as simple (AO/OTA types B1-3) or complex (AO/OTA types C1-3). For analysis, there were nine simple and three complex fracture patterns.

**Fig. 2:**
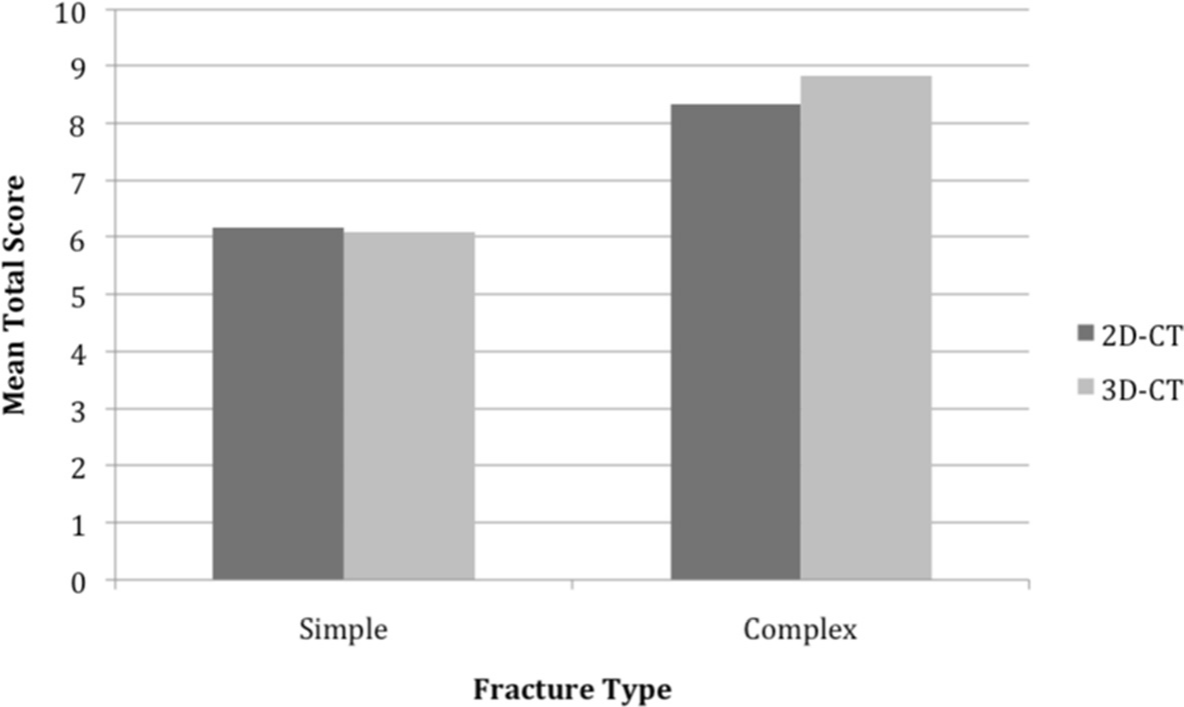
Mean total scores based on fracture complexity and plan type

When combining all cases (2D- and 3D-CT) and comparing complex to simple fractures, there is a significant difference in mean total scores (6.15 *vs.* 8.63, *p* < 0.0001). Comparing complex to simple fractures between plan types demonstrates no significant differences (*p* = 0.443) (Fig. 2).

## Discussion

In trauma surgery, preoperative planning is of the utmost importance, and can determine the success of a procedure [11, 15]. The surgeon must plan their surgical approaches, the steps of reduction, the placement of hardware, and the need for any adjuncts such as bone graft. Planning also ensures one has all of the essential tools and materials at their disposal [15].

The role of 2D-CT in the preoperative management of tibial plateau fractures is rarely debated. Chan *et al.* have demonstrated that the addition of 2D-CT to plain film radiography influenced the preoperative plan for tibial plateau fractures 26 % of the time. The main reason for the significant changes in management plans was stated to be due to unappreciated comminution on the plain film images [13].

Several authors have investigated the utility of 3D-CT reconstructions in orthopaedic trauma surgery. The rationale for using 3D-CT is that it may help surgeons understand the fracture pattern better, and therefore aid in classifying and managing the fractures. Many authors have stated the importance of understanding the fracture pattern to achieve the surgical goals of anatomic reduction and stable fixation with minimal soft tissue disruption [1, 2, 11, 15].

In the present study, we compared plans made based on plain radiographs and 2D-CT (the current standard) to plans made with the addition of 3D-CT reconstructions. Observers did not have access to their initial plans when making plans with the 3D reconstructions.

We were unable to demonstrate any significant change in preoperative plans when comparing 2D- to 3D-CT. The level of training of the observer and the fracture complexity did not affect this result. Although we could detect statistically significant differences with regards to certain aspects of the preoperative plans based on level of training (incision and implants), the small differences are not clinically significant. Staff surgeons’ plans had the largest change between plan types, however this change only represented 0.24 points. This change is too small to have a significant clinical impact, given that at least one point is necessary to have even a minor change in the overall preoperative plan. Senior residents were the only group to have a higher score on their 2D-CT preoperative plans. This suggests they tend to either overestimate the severity of the injury on 2D-CT, or underestimate the severity of injury on 3D-CT. This is in contrast to the staff surgeons, who had a higher point score on their 3D-CT preoperative plans. Further investigation with a larger number of complex fractures could help delineate this finding. The change in arthrotomy rates observed in junior and senior residents plans with the addition of 3D-CT is difficult to interpret, given that there are no absolute indications for arthrotomy in this situation. Subjective responses from observers confirmed that the addition of 3D-CT reconstructions was of limited benefit when making preoperative plans for these 12 cases.

There are several limitations of this study. The first is the use of a preoperative planning tool that has not been previously validated, however such a tool does not exist in the literature. This tool was created by consensus and expert opinion of several orthopaedic trauma surgeons at a level I trauma centre. Although it does not include all aspects of the surgical plan for every tibial plateau fracture one may encounter, we believe it includes the pertinent details for the most common tibial plateau fracture patterns treated in our centre. The use of a point-based system greatly simplifies data analysis of this type of study. Although the point totals, such as the mean total score, do not describe where the points come from, our subanalysis demonstrated no significant changes of any subset of the plan when comparing 2D-CT to 3D-CT. In addition, we think that it is very unlikely that based on a 3D-CT scan, a surgeon or resident would change their plan from an anterolateral incision and lateral implants to a posteromedial incision and medial or posterior implants. The second weakness is the small number of ‘complex’ tibial plateau fractures included. The majority of the fractures were classified as Shatzker II. It is possible that a larger number of Shatzker V and VI fractures would influence the outcome of this study. In our opinion, however, this distribution of fracture patterns accurately describes the complexity of tibial plateau fractures commonly seen at our centre. This study, then, demonstrates a realistic assessment of the utility of 3D-CT in our patient population. Finally, we did not review postoperative radiographs or operative reports to compare them to the preoperative plans. This step could have shed light on our preoperative planning tool’s accuracy. We do not believe that we would be able to detect differences in comparing the 2D-CT and 3D-CT preoperative plans to the final operative report. The small differences noted between the two plan types are likely too small to represent a change in the final operative construct. However, without having analyzed this specifically it represents a limitation in our study.

The cost of 3D-CT over 2D-CT is an issue that should be addressed. Before the routine addition of any diagnostic modality, it should be evaluated thoroughly to ensure its usefulness justifies its cost. The current literature quotes an increase in cost of 20 % when 3D-CT reconstructions are added to a standard 2D-CT scan [6, 8]. In our centre, the increase in cost is an estimated $200 per study. With an estimated 35 tibial plateau fractures assessed at our level I trauma centre per year (which is only one of four adult centers in our city), there would be an increased cost of $7000 per year if 3D-CT reconstructions were routinely used. The current study suggests that this cost may not be justified for routine use for all tibial plateau fractures. Further investigation must be done to determine if this cost is justified in the setting of more complex (type C) tibial plateau fractures, as our study did not address these adequately. In addition, with easier access to free software to perform these reconstructions, cost may not play a significant role in their use.

## Conclusions

The use of three-dimensional computed tomography has been under increasing investigation over the past several years. Few authors have investigated the impact that these reconstructions have on fracture management. All new diagnostic modalities come at a cost, and therefore should be evaluated prior to their widespread use. Our study demonstrated no significant influence on the preoperative plans of tibial plateau fractures when 3D-CT was added to 2D-CT. Given the added cost, we question its utility when managing this particular fracture pattern. Further investigation into the use of 3D-CT with regards to other intra-articular fractures will help to identify the best use of this imaging technique.

## Abbreviations

CT: Computed tomography
2D-CT: Computed tomography with coronal, sagittal, and axial reconstructions
3D-CT: Computed tomography with three-dimensional reconstructions
